# Gut microbiota contributes to the development of endometrial glands in gilts during the ovary-dependent period

**DOI:** 10.1186/s40104-021-00578-y

**Published:** 2021-05-05

**Authors:** Baoyang Xu, Wenxia Qin, Yiqin Yan, Yimei Tang, Shuyi Zhou, Juncheng Huang, Chunlin Xie, Libao Ma, Xianghua Yan

**Affiliations:** 1 State Key Laboratory of Agricultural Microbiology, College of Animal Sciences and Technology, Huazhong Agricultural University, Wuhan, 430070 Hubei China; 2 The Cooperative Innovation Center for Sustainable Pig Production, Wuhan, 430070 Hubei China; 3 Hubei Provincial Engineering Laboratory for Pig Precision Feeding and Feed Safety Technology, Wuhan, 430070 Hubei China

**Keywords:** Endometrial gland, Fecal microbiota transplantation, Gut microbiota, Meishan gilts, Steroid hormones, Untargeted metabolomics, Uterus

## Abstract

**Background:**

The hyper-prolificacy Meishan gilts achieved a superior endometrial gland development (EGD) than white crossbred gilts during the ovary-independent period (before 60 d of age). Then, the EGD continues under the management of ovary-derived steroid hormones that regulated by gut microbiota (after 60 d of age). However, whether Meishan gilts’ superiority in EGD lasting to the ovary-dependent period (after 60 d of age) and the role of gut microbiota in this period both remain unclear.

**Methods:**

Meishan gilts and Landrace x Yorkshire (LxY) gilts were raised under the same housing and feeding conditions until sexual maturity and then we compared their EGD and gut microbiota. Meanwhile, we transplanted fecal microbiota from Meishan gilts to L×Y gilts to explore the role of gut microbiota in EGD. We sampled plasma every 3 weeks and collected the uterus, ovary, liver, and rectal feces after the sacrifice. We then determined the hormone concentrations and expressions of the EGD-related genes. We also profiled the gut microbiota using 16S rDNA sequencing and metabolites of plasma and liver tissue using untargeted metabolomics. Finally, the correlation analysis and significant test was conducted between FMT-shifted gut microbes and EGD-related indices.

**Results:**

Meishan gilts have larger endometrial gland area (*P* < 0.001), longer uterine horn length (*P* < 0.01) but lighter uterine horn weight (*P* < 0.05), a distinctive gut microbiota compared with L×Y gilts. Fecal microbiota transplantation (FMT) increased endometrial gland area (*P* < 0.01). FMT markedly shifted the metabolite profiles of both liver and plasma, and these differential metabolites enriched in steroid hormone biosynthesis pathway. FMT increased estradiol and insulin-like growth factor 1 but decreased progesterone dynamically. FMT also increased the expression of the EGD-related genes estrogen receptor 1 gene, epithelial cadherin, and forkhead box protein A2. There is a significant correlation between FMT-shifted gut microbes and EGD-related indices.

**Conclusion:**

Sexually matured Meishan gilts achieved a superior EGD than LxY gilts. Meanwhile, gut microbiota contribute to the EGD potentially via regulating of steroid hormones during the ovary-dependent period.

**Supplementary Information:**

The online version contains supplementary material available at 10.1186/s40104-021-00578-y.

## Background

Mammalian uteri contain glands in the endometrium that synthesize or transport and secrete substances essential for survival and development of the conceptus (embryo/fetus and associated extraembryonic membranes), thus contributing to female prolificacy [1, 2]. Endometrial adenogenesis is primarily a postnatal event in sheep, pigs, and rodents [3]. At birth, no endometrial glands are present in uterus and then within 7 d, endometrial glands begin to differentiate, and they continue to differentiate during the next several months [3]. Neonatal porcine uterine development occurs in an ovary-independent manner before, and in an ovary-dependent manner after, day 60 of neonatal life [4, 5]. In ovary-dependent development period, ovarian hormones estradiol and progesterone play crucial roles in endometrial glands development [5]. Estradiol is a primary mitogen for uterine epithelium and its increase around estrous induces proliferation of both uterine luminal epithelium and glandular epithelium [5]. Neonatal estradiol exposure alters uterine morphology and endometrial transcriptional activity in prepubertal gilts [6]. Progesterone can inhibit estradiol actions in stimulation of uterine epithelial proliferation through epithelial progesterone receptors [5, 7]. This relationship between estradiol and progesterone may regulate the endometrial gland development and uterine function.

Meishan pigs as a Chinese indigenous breed is characterized by its prolificacy, producing an average of three to four more piglets per litter than European breeds [8]. However, the underlying mechanism by which Meishan pigs achieve superior litter size remains not fully understood. Christenson et al. found that Meishan gilts’ uterus secreted more abundance of endometrial proteins, which increased in association with endometrial gland development before day 60 of neonatal life [9]. Further histological analysis validated that Meishan gilts achieved a superior endometrial gland development than white crossbred gilts at 60 d of age [9]. However, the further comparative morphological study of their endometrial gland development after 60 d of age remains undetermined. A recent study showed that the Meishan sows of high-prolificacy both have increased fecal microbiota diversity and fecal steroid hormones estradiol and progesterone levels than low-prolificacy ones, which may contribute to the improvement of sows’ reproductive performance [10]. Interestingly, microbiota that harbor inside the host intestine function as a vital endocrine organ get involved in regulating steroid hormones estradiol and progesterone [11, 12].

Given that the vital role of steroid hormones estradiol and progesterone in endometrial glands development during the ovary-dependent period [7, 13, 14], we hypothesis that gut microbiota get involved in the superior endometrial gland’s development of prolific Meishan gilts than white crossbred gilts during the ovary-dependent period. Fecal microbiota transplantation (FMT) involves the transfer of donor fecal microbiome to a recipient in order to establish a more desirable or donor-like gut microbiome [15, 16]. FMT provides an effective approach to link gut microbiota and host phenotype [15, 17]. To test our hypothesis, we compared the uterine development and gut microbiota between sexually matured Meishan gilts and crossbred Landrace × Yorkshire (L×Y) gilts under the same housing and feeding conditions. Meanwhile, for exploring the role of gut microbiota in endometrial glands development, we transplanted fecal microbiota of Meishan gilts to recipient  L×Y gilts.

## Methods

### Experimental design and animals

All experiments involving swine were carried out under the recommendations of the Guide for the Care and Use of Laboratory Animals Monitoring Committee of Hubei Province, China, and the protocol was approved by the Scientific Ethics Committee of Huazhong Agricultural University (approval numbers HZAUSW-2018-015). All efforts were made to minimize animal suffering. Eight Meishan gilts of age 95 ± 7 d from the National Conservation Farm of Meishan Pigs (Jiading, Shanghai) and 14 Landrace × Yorkshire (L×Y) gilts of age 90 ± 4 d from a commercial farm were housed in National Engineering and Technology Research Center for Livestock and fed with diets according to NRC (2012). Experimental design: L×Y gilts receive 20 mL saline (Ctrl) or fecal suspension from Meishan gilts (FMT) and these Meishan gilts receive the same volume of saline (MS) every other day until the puberty onset. For reducing the anaerobic bacteria die, we prepared fecal slurry with O_2−_free saline immediately after collecting fresh feces. We then regulated the concentration of fecal slurry after counting the live microbes using optical microscopy combined with methylene blue staining [18]. All gilts were sacrificed at 24 h after detection of the third estrus (excluded a Meishan gilts because of its health concerns). Blood samples (10 mL) were collected and then centrifugate for 10 min at 3,500×*g* at 4 °C to get plasma samples. Immediately after slaughter, intestinal contents from rectum were collected and placed in liquid nitrogen. We determined the weight and length of uterine horn. Uterus tissue was removed from the middle portion of the uterus and rinsed with iced cold PBS, then fixed in 4% paraformaldehyde solution for measuring uterus morphology or placed in liquid nitrogen.

### Fecal microbiota transplantation (FMT) experiment

The fecal suspension was prepared using the protocol previously described with minor optimization [19]. Briefly, fresh feces samples were obtained from Meishan gilts and immediately homogenized in sterile and O_2_-free saline solution. Then fecal slurry was passed through the sterilized gauze and then a 0.224-mm stainless cell strainer to remove the particles. We used optical microscopy combined with methylene blue staining to count the live microbes in the fecal slurry. The Meishan gilts’ neighbor L×Y gilts received sterile saline (20 mL) or fecal microbiota suspensions (10^7^ CFU/mL, 20 mL) every other day from the age of 90 d to the age of puberty onset (about 200 d of age).

### Evaluation of endometrial gland development

We evaluated endometrial gland development by examining hematoxylin-and eosin-stained (H&E) uterine wall sections (10 μm) using computer-assisted morphometry. For each section, six areas of the endometrium from lumen to myometrium were randomly selected and measured. Then, the total area of glands within the endometrial area was measured by encircling each gland within the previously outlined endometrial area. Gland development for that section was then expressed as the ratio of gland area to endometrial area [20].

### Gut microbiota profiling

The total genomic DNA of fecal bacteria (stool from the rectum) was extracted using the protocol of the Repeated Bead Beating Plus Column Method [21]. The integrity of DNA was assessed by agarose gel electrophoresis. The genomic DNA was used as a template for PCR amplification. Universal primers 338F and 806R were used for PCR amplification of the V3–V4 hypervariable regions of 16S rRNA genes (338F, 5′-ACTCCTACGGGAGGCAGCA-3′, 806R, 5′-GGACTACHVGGGTWTCTAAT-3′) [22]. The generated DNA pool was then sequenced on the Illumina HiSeq system with the sequencing strategy PE300 and then the sequencing data were analyzed using the Quantitative Insights Into Microbial Ecology software package [23].

To obtain more accurate and reliable results in subsequent bioinformatics analysis, raw data were cleansed by the in-house procedure [22]. Then paired end reads with overlap were merged to tags. The high-quality paired-end reads were combined to tags based on overlaps with FLASH [24]. The tags were clustered to OTU (Operational Taxonomic Unit) by scripts of software USEARCH(v7.0.1090, [25]). After that, they were clustered into OTU with a 97% threshold using UPARSE, and the OTU unique representative sequences were obtained; Chimeras were filtered out by UCHIME(v4.2.40); OTU representative sequences were taxonomically classified using Ribosomal Database Project (RDP) Classifier v.2.2 trained on the database Greengene_2013_5_99 [26] with 0.6 confidence values as cutoff. The alpha diversity indices including observed species value, Chao1 value, ACE value, Shannon, and Simpson value are calculated by Mothur (v1.31.2, [27]) with the corresponding rarefaction curve was drawn by software R (v3.6.0). Phylogenetic beta diversity measures such as unweighted UniFrac distance metrics analysis and principal-component analysis (PCoA) were done using the QIIME 2 [23]. Functional analysis of gut microbiota was predicted by Phylogenetic Investigation of Communities by Reconstruction of Unobserved States 2 (PICRUSt2, [28]).

### Gut microbial metabolites

We determined the microbial Metabolites short-chain fatty acids (SCFAs) (acetate, propionate, butyrate) in the diegesta of colon using gas chromatography with a modification of the previous method [29]. In brief, 1 g of the digesta samples was weighed into a 2-mL centrifuge tube with 1 mL of methanol added. After being vortexed for 30 s, the sample was centrifuged (12,000 × *g*) at 4 °C for 10 min. The supernatant was transferred into centrifuge tubes (2 mL) and mixed with 0.2 mL 25% metaphosphoric acid. After 30 min at 4 °C, the tubes were centrifuged (12,000 × *g*) again at 4 °C for 10 min. To quantify SCFAs, a calibration curve for the concentration range of 0.015–1 mg/mL was constructed. SCFAs measurements were performed following a recently published protocol [30].

### Quantitative enzyme-linked immunosorbent assay (ELISA)

Steroid hormones estradiol, progesterone, and insulin-like growth factor 1(IGF-1) in plasma and tissue were quantified by ELISA (Cusabio, Wuhan, China). We collected plasma samples using EDTA as an anticoagulant and then the samples were centrifuged for 15 min at 1,500 × *g* and 4 °C. For tissue homogenates, 100 mg tissue was rinsed with 1× PBS, homogenized in 1 mL of 1× PBS and stored overnight at − 20 °C. After two freeze-thaw cycles were performed to break the cell membranes, the homogenates were centrifuged for 5 min at 5,000 × *g*, and 4 °C. The supernatant was removed and assayed immediately. Alternatively, aliquot and store samples at − 20 °C or − 80 °C. Centrifuge the sample again after thawing before the assay. Avoid repeated freeze-thaw cycles. All samples were measured in 3 replicates following the recommended procedures in the instruction.

### Quantitative real-time PCR

qPCR was performed with the PowerUp™ SYBR™ Green Master Mix (Applied Biosystems) on a CFX384 Real-Time PCR system (Bio-Rad) and changes in gene expression were calculated relative to *GAPDH*. RT-qPCR primer pairs are: estrogen receptor 1 gene (*ESR1*) 5′-AGCACCCTGAAGTCTCTGGA-3′, 5′-TGTGCCTGAAGTGAGACAGG-3′; epithelial cadherin (*CDH1*) 5′- ATGTGCACGTATGCGACTGT-3′, 5′-GGAACTTGCAATCCTGCTTC-3; forkhead box protein A2 (*FOXA2*) 5′ -ATGCTGGGAGCGGTGAAGAT-3′, 5′-AGCGAGTGGCGGATGGAGTT-3′; glyceraldehyde-3-phosphate dehydrogenase (*GAPDH)* 5′-T CGGAGTGAACGGATTTGGC-3′, 5′-TGCCGTGGGTGGAATCATAC-3′.

### Hematoxylin and eosin (H&E) staining

Briefly, ovaries were preserved in 4% paraformaldehyde overnight at 4 °C (Sigma-Aldrich). The tissues were subsequently embedded in paraffin wax (Fisher Scientific) following immersion in a graded series of alcohols (70–100%). Embedded tissue was sectioned (5 mm) using a rotary microtome. We adhered these sections to microscope slides and then dried them at 56 °C for 24 h. Next, we passed slides through a series of the clearing agent xylene and rehydrated in a graded series of ethanol (100%, 100%, 100%, 95%, 80%). After a brief wash in distilled water, we incubated the slides with hematoxylin solution. The sections were then washed with running tap water to remove excess hematoxylin. Then, we differentiated the sections in 1% acid alcohol for 30 s and then washed them with running tap water for 1 min. This step was followed by an incubation in the eosin counterstain, subsequent dehydration in a graded series of ethanol (80%, 95%, 95%, 95%, 100%, 100%, 100%), and immersion in xylene.

### Untargeted metabolomics profiling

Sample preparation. Plasma metabolite extraction was performed by mixing 40 μL thawed plasma and 120 μL ice-cold methanol, vortexing for 1 min, 10 min standing at room temperature, and keeping at − 20 °C for 2 h. After centrifuged at 4,000 × *g* and 4 °C for 30 min, 25 μL of the supernatant was transferred into a glass vial containing 225 μL of 50% methanol. Take 50 μL of each sample and mix it into a quality control (QC) sample and divide all samples into 96-well plates in the order of loading and put 60 μL in each well. Liver metabolite extraction was performed by mixing 25 mg thawed liver tissue and 800 μL of chilled methanol/water (1:1) solution. Add two small steel balls to each EP tube, place the sample in TissueLyser and grind. The parameter is set to 50 Hz for 3 min. After grinding, remove the steel ball, place the EP tube in the − 20 °C refrigerator for 2 h. After centrifuged at 30,000 × *g* and 4 °C for 15 min, carefully take 650 mL of each sample into a new EP tube. Then centrifuged at 25,000 × *g* and 4 °C for 20 min, take 550 mL of each sample in a new EP tube. Take 1 mL of acetonitrile through the column with a positive pressure extractor, then take 1 mL of 100% methanol through the column at a constant rate, and finally take 1 mL of Milli-Q pure water through the column at a constant rate. Draw 400 mL supernatant after centrifugation for each sample, transfer to the activated and equilibrated SPE column, after repeating once, discard the supernatant, add 400 mL 100% acetonitrile to elute, keep eluted solution in a new EP tube. Take 40 μL of all samples and mix them into QC. Dispense all the samples into 96-well plates with 60 μl of each sample in each well.

Metabolomics analysis. For plasma and liver tissue sample, all samples were gained by the Liquid Chromatography Mass Spectrometry (LC-MS) system followed machine orders. First, all chromatographic separations were performed using an ultra-performance liquid chromatography (UPLC) system (Waters, UK). For the reversed phase separation, plasma sample used an ACQUITY UPLC BEH C18 column (100 mm × 2.1 mm, 1.7 μm, Waters, UK) and liver tissue sample used an ACQUITY UPLC HSS T3 column (100 mm × 2.1 mm, 1.8 μm, Waters, UK). The column oven was maintained at 50 °C. The flow rate was 0.4 mL/min and the mobile phase comprised solvent A (water + 0.1% formic acid) and solvent B (acetonitrile + 0.1% formic acid). Gradient elution conditions were set: 0–2 min, 100% phase A; 2–11 min, 0 to 100% B; 11–13 min, 100% B; 13–15 min, 0 to 100% A. The injection volume for each sample was 10 μL. A high-resolution tandem mass spectrometer Xevo G2 XS QTOF (Waters, UK) was used to detect metabolites eluted form the column. The Q-TOF was operated in both positive and negative ion modes. For positive ion mode, the capillary and sampling cone voltages were set at 3.0 kV and 40.0 V, respectively. For negative ion mode, the capillary and sampling cone voltages were set at 2.0 kV and 40.0 V, respectively. The mass spectrometry data were gained in Centroid MSE mode. The TOF mass range was from 50 to 1200 Da and the scan time was 0.2 s. For the MS/MS detection, all precursors were fragmented using 20–40 eV, and the scan time was 0.2 s. During the acquisition, the LE signal was gained every 3 s to calibrate the mass accuracy. In order to test the stability of the LC-MS during the whole acquisition, a quality control sample (Pool of all samples) was gained after every 10 samples. Peak extraction is mainly achieved through the commercial software Progenesis QI (version 2.2), including peak alignment, peak extraction, normalization, deconvolution, and compound identification. Based on QC sample information, local polynomial regression fitting signal correction (Quality control–based robust LOESS signal correction, QC-RSC) is performed on the real sample signal [31].

### Statistical analysis

Results were presented as mean ± SEM. Experimental data were analyzed by one-way analysis of variance tests, followed by Fisher’s least significant difference and the Duncan multiple comparison test with GraphPad 8.0 software. Significance was presented as *P < 0.05, and **P < 0.01, whereas *P* values between 0.05 and 0.10 were considered as indicative of a trend.

## Results

### Meishan gilts have distinct uterine characters and gut microbiota compared with LxY gilts

**Fig. 1:**
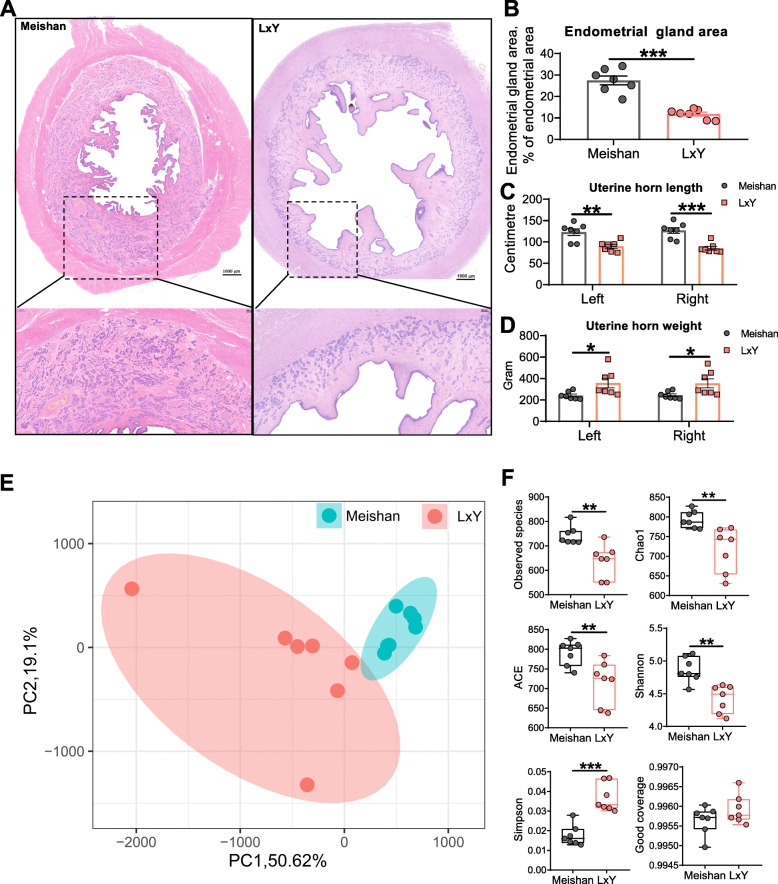
Meishan gilts have distinct uterine characters and gut microbiota compared with LxY gilts. **a** Representative H&E-stained uterine sections. Scale bar, 1,000 μm and 200 μm. **b** Endometrial gland area normalized to endometrial area. Length (**c**) and weigh (**d**) of Uterine horn. **e** PCoA based on weighted UniFrac distance show a distinct fecal microbial composition inter-groups. Each data point represents a sample from a distinct sow projected onto the first two principal coordinates (percent variation explained by each PCo is shown in parentheses). **f** Species diversity analysis of gut microbiota with alpha diversity indices including Observed species, Chao1, ACE, Shannon, Simpson and Good coverage. *P < 0.05, **P < 0.01, ***P < 0.001, data were shown as means ± SEM; *n* = 7

Under the same housing and feeding conditions, Meishan gilts have larger (*P* < 0.001) endometrial gland area normaled to the endometrial area (Fig. 1a-b) and longer (*P* < 0.01 for left uterine horn and *P* < 0.001 for right uterine horn) uterine horn length (Fig. 1c) but lighter (*P* < 0.05) uterine horn weight (Fig. 1d) compared with L×Y gilts at the age of third estrus. The beta diversity of gut microbiota showed by PCoA showed obvious difference between Meishan gilts and L×Y gilts (Fig. 1e). Meishan gilts had superior alpha diversity indices including observed species (*P* < 0.01), Chao1 (*P* < 0.01), ACE (*P* < 0.01), Shannon (*P* < 0.01) and Simpson (*P* < 0.001) compared with L×Y gilts (Fig. 1f). The alpha diversity index Good coverage suggested that all the sequencing data were of high quality and could cover the most species. Further taxon analysis showed that Meishan gilts’ gut harbored more bacteria of phyla Actinobacteria (*P* < 0.01), Firmicutes (*P* < 0.01), Fibrobacteres (*P* < 0.001), Lentisphaerae (*P* < 0.05), and Synergistetes (*P* < 0.01) but fewer bacteria of phyla Bacteroidetes (*P* < 0.001), and WPS-2 (*P* < 0.01) compared with L×Y gilts (Supplemental Figure S1a). At genus level, Meishan gilts’ gut harbored more *Bacteroides* (*P* < 0.01), *Bifidobacterium* (*P* < 0.001), *Fibrobacter* (*P* < 0.001), *Oscillospira*(*P* < 0.05), *SMB53*(*P* < 0.05), and Turicibacter (*P* < 0.05) but less *Anaerovibrio* (*P* < 0.05), *Bulleidia* (*P* < 0.01), *Butyricicoccus* (*P* < 0.01), *Coprococcus* (*P* < 0.05), *Dorea* (*P* < 0.05), *Gemmiger* (*P* < 0.05), *Lachnospira* (*P* < 0.01), *Pseudobutyrivibrio* (*P* < 0.01), and *Streptococcus* (*P* < 0.05) compared with L×Y gilts (Supplemental Figure S1b).

### FMT shifted gut microbiota and increased the endometrial gland area in recipient LxY gilts

**Fig. 2:**
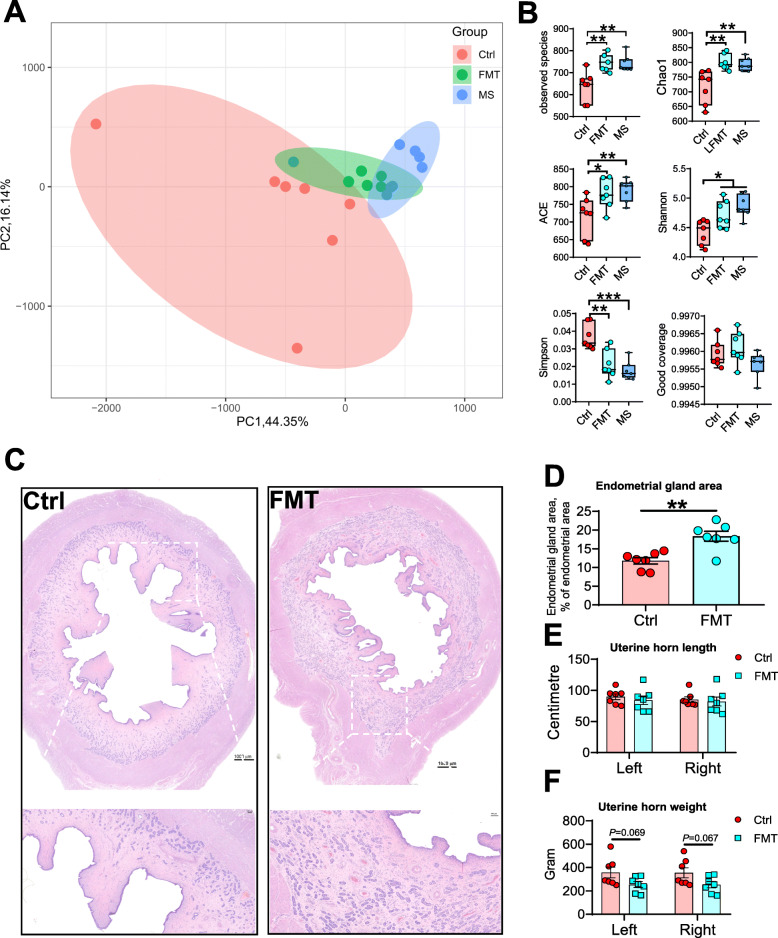
FMT shifted gut microbiota and increased the endometrial gland area in recipient L×Y gilts. **a** PCoA based on weighted UniFrac distance show a distinct fecal microbial composition intergroups. Each data point represents a sample from a distinct sow projected onto the first two principal coordinates (percent variation explained by each PCo is shown in parentheses). **b** Species diversity analysis of gut microbiota with alpha diversity indices including Observed species, Chao1, ACE, Shannon, Simpson and Good coverage. **c** Representative H&E-stained uterine sections. Scale bar, 1,000 μm and 200 μm. **d** Endometrial gland area normalized to endometrial area. Length (**e**) and weigh (**f**) of Uterine horn. *P < 0.05, **P < 0.01, ***P < 0.001, data were shown as means ± SEM; *n* = 7

**Fig. 3:**
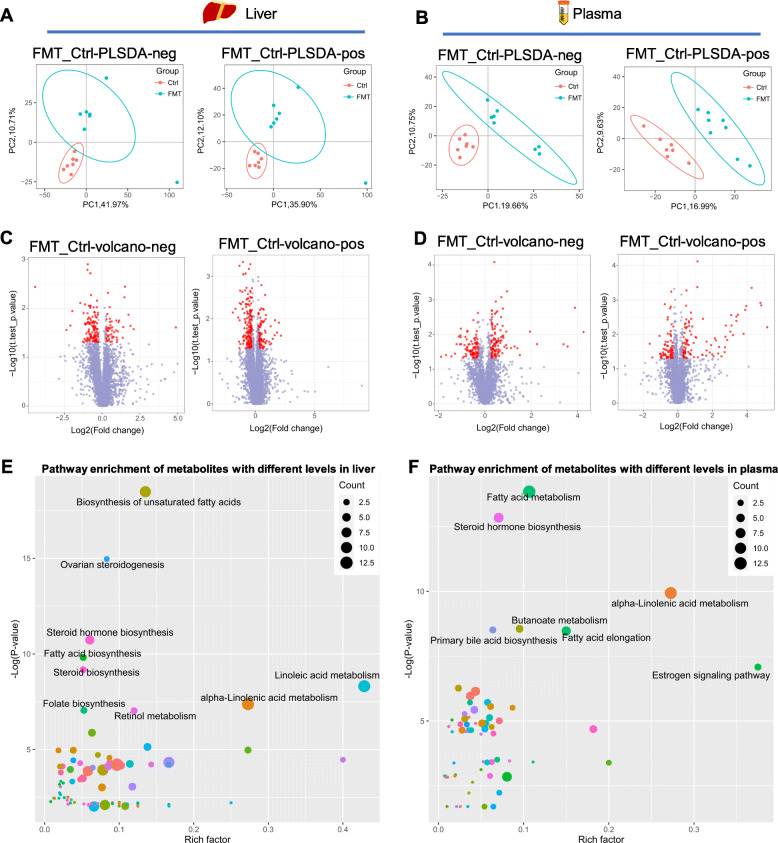
FMT shifted the metabolite profiles of liver and plasma in recipient L×Y gilts. PLS-DA of the detected metabolites of both pos and neg mode in liver (**a**) and plasma (**b**). The volcano plots of the significantly differential metabolites (red dot) under both pos and neg mode in liver (**c**) and plasma (**d**). KEGG pathway enrichment analysis for the different metabolites in both liver (**e**) and plasma (**f**). *n* = 7

To explore the association between the gut microbiota and uterine development, we transplanted the fecal microbiota of Meishan gilts to L×Y gilts. PCoA showed that FMT distinctly shifted the gut microbiota composition of the recipient L×Y gilts and made it closed to that of Meishan gilts (Fig. 2a). The top two principal-component (PC1 and PC2) suggested that FMT explained 60.49% of the variation (Fig. 2a). FMT recipient gilts had superior alpha diversity indices including observed species (*P* < 0.01), Chao1 (*P* < 0.01), ACE (*P* < 0.05), Shannon (*P* < 0.05) and Simpson (*P* < 0.01) compared with control L×Y gilts (Fig. 2b). The alpha diversity index Good coverage suggested that all the sequencing data were of high quality and could cover the most species. Further taxon analysis showed that FMT recipient gilts’ gut harbored more bacteria of phyla Firmicutes (*P* < 0.001), Fibrobacteres (*P* < 0.001), but fewer bacteria of phyla Bacteroidetes (*P* < 0.001) compared with control L×Y gilts (Supplemental Figure S2a). At genus level, FMT recipient gilts’ gut harbored more *Bifidobacterium* (*P* < 0.01) and *Fibrobacter* (*P* < 0.01) compared with control L×Y gilts (Supplemental Figure S2b). Morphological analysis(H&E) of uterine showed that FMT recipient L×Y gilts had increased (*P* < 0.01) endometrial gland area (Fig. 3c-d). FMT had no effect on the uterine horn length (Fig. 3e) but had a trend (*P* = 0.069 for left uterine and *P* = 0.067 for right uterine) to reduce the uterine horn weight (Fig. 3f).

### Untargeted metabolomics revealed metabolism shift induced by FMT in liver and plasma

To link the shifted gut microbiota and increased endometrial gland area, we determined the metabolites profiles of liver and plasma using untargeted metabolomics. Partial least squares discriminant analysis (PLS-DA) showed that the detected metabolites of both pos and neg mode were obviously shifted by FMT in liver (Fig. 3a) and plasma (Fig. 3b). The volcano plots showed the significantly differential metabolites (red dot) under both pos and neg mode in liver (Fig. 3c) and plasma (Fig. 3d). We further conducted KEGG pathway enrichment analysis for the different metabolites in both liver and plasma. For liver, the FMT-induced differential metabolites enriched in pathways of steroid hormone biosynthesis, steroid biosynthesis, ovarian steroidogenesis, linoleic acid metabolism, folate biosynthesis, and biosynthesis of unsaturated fatty acids (Fig. 3e). And for plasma, the FMT-induced differential metabolites enriched in pathways of steroid hormone biosynthesis, primary bile acid biosynthesis, fatty acid metabolism, fatty acid degradation, estrogen signaling pathway, butanoate metabolism, and alpha-Linolenic acid metabolism (Fig. 3f). Noteworthily, steroid hormone biosynthesis pathway was enriched by FMT-induced differential metabolites from both liver and plasma, which indicated that the circulating steroid hormone profile has been shifted by FMT.

### FMT shifted endometrial gland development-related hormones in recipient LxY gilts

**Fig. 4:**
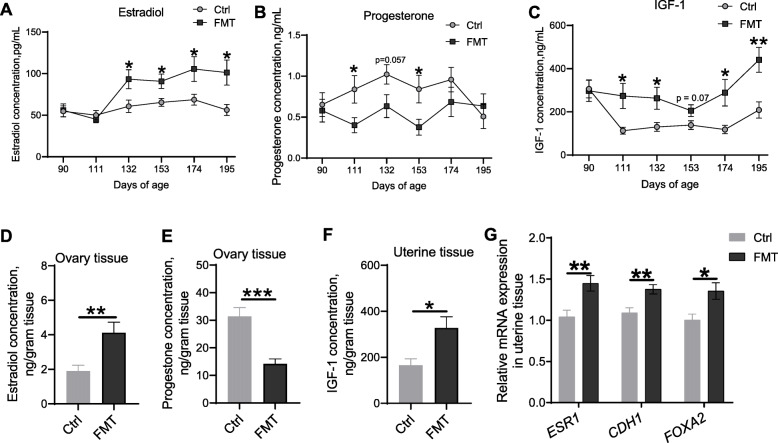
FMT shifted uterine gland development-related hormones and gene expression in recipient LxY gilts. Dynamic shifts of uterine gland development-related hormones estradiol (**a**), progesterone (**b**), and IGF-1 (**c**). The concentrations of estradiol (**d**) and progesterone (**e**) in ovary tissue; and IGF-1 concentration (**f**) in uterine tissue. The relative mRNA expression of endometrial gland development-related genes (**g**). *P < 0.05, **P < 0.01, ***P < 0.001, data were shown as means ± SEM; *n* = 7

Given that FMT shifted the circulating steroid hormone profile, we then determined the concentration of steroid hormones estradiol and progesterone, and IGF-1 in plasma. FMT increased plasma estradiol concentration on 132(*P* < 0.05), 153(*P* < 0.05), 174(*P* < 0.05), and 195(*P* < 0.05) d of age (Fig. 4a), but decreased plasma progesterone concentration on 111(*P* < 0.05), 132(*P* = 0.057), and 153(*P* < 0.05) d of age (Fig. 4b). Estradiol and progesterone can regulate of IGF-1 gene expression in pig uterus [32]. We therefore determined the IGF-1 concentration in plasma and uterine tissue. The results showed that FMT increased IGF-1 on 111(*P* < 0.05), 132(*P* < 0.05), 153 (*P* = 0.07), 174(*P* < 0.05), and 195(*P* < 0.01) d of age (Fig. 4c), and in uterine tissue(*P* < 0.05) (Fig. 4f). The enhanced secretion of estradiol (*P* < 0.01) and progesterone(*P* < 0.001) were determined in ovary tissue (Fig. 4d-e). Correspondingly, FMT elevated (*P* < 0.01) the mRNA expression level of estrogen receptor 1 gene (*Esr1*) (Fig. 4g). Besides, FMT elevated the mRNA expression level of uterine development -related epithelial cadherin (*CDH1*) (*P* < 0.01) and forkhead box protein A2 (*FOXA2*) (*P* < 0.05) (Fig. 4g).

### FMT promoted carbohydrate metabolism and digestive system functions of gut microbiota in recipient LxY gilts

**Fig. 5:**
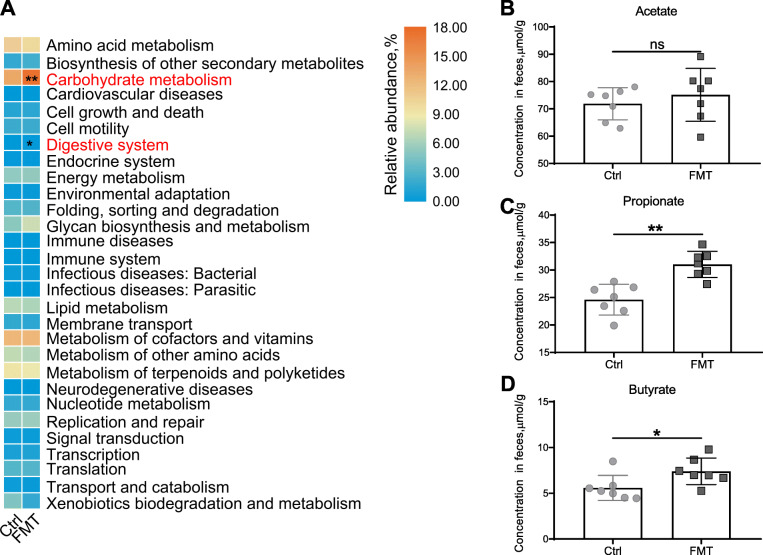
FMT promoted carbohydrate metabolism and digestive system functions of gut microbiota in recipient L×Y gilts. KEGG functions of gut microbiota (**a**) and the corresponding concentrations of SCFAs (**b**). The differential KEGG function was indicated in red. *P < 0.05, **P < 0.01, data were shown as means ± SEM; *n* = 7

To explore the underlying mechanism by which the shifted gut microbiota contributes to the endometrial gland development, we determined the function shift of gut microbiota using PICRUSt2. The results suggested that FMT enriched KEGG functions of carbohydrate metabolism (*P* < 0.01) and digestive system (*P* < 0.05) in gut microbiota of recipient L×Y gilts (Fig. 5a). This enrichment effect of FMT was confirmed by the increased concentrations of SCFAs propionate (*P* < 0.01) and butyrate (*P* < 0.05) but not acetate (Fig. 5b-d).

### FMT-shifted gut microbes were correlated to the indices of uterine development

**Fig. 6:**
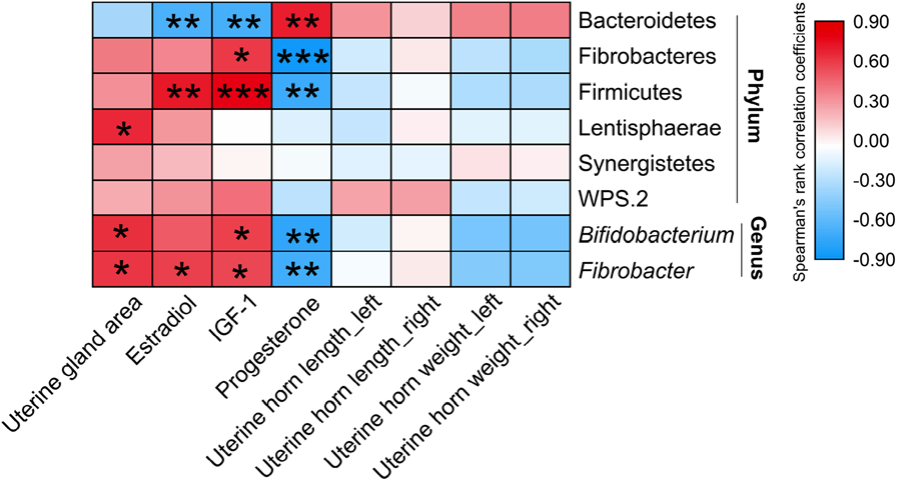
FMT-shifted gut microbes were correlated to the indices of uterine development. The correlation between FMT-shifted gut microbes and uterine development-related indices using spearman’s rank correlation coefficient and significance test (*n* = 7). *P < 0.05, **P < 0.01, ***P < 0.001; *n* = 7

We finally determined the correlation between FMT-shifted gut microbes and uterine development-related indices using spearman’s rank correlation coefficient and significance test. The results in Fig. 6 showed that Lentisphaerae(*P* < 0.05), *Bifidobacterium*(*P* < 0.05), and *Fibrobacter*(*P* < 0.05) were positively correlated to endometrial gland area. Firmicutes(*P* < 0.01) and *Fibrobacter*(*P* < 0.05), were positively correlated to estradiol concentration; Bacteroidetes was significantly negatively correlated to estradiol (*P* < 0.01). For IGF-1, Bacteroidetes was negatively correlated to IGF-1 concentrations(*P* < 0.01); Fibrobacteres(*P* < 0.05), Firmicutes(*P* < 0.001), *Bifidobacterium*(*P* < 0.05), and *Fibrobacter*(*P* < 0.05) were positively correlated to IGF-1 concentration. For progesterone, Bacteroidetes was positively correlated to progesterone concentration(*P* < 0.01); Fibrobacteres(*P* < 0.001), Firmicutes(*P* < 0.01), *Bifidobacterium*(*P* < 0.01), and *Fibrobacter* (*P* < 0.01) were negatively correlated to progesterone concentration.

## Discussion

Porcine uterine development plays a crucial role in determining sows’ prolificacy [4, 5]. Earlier evidences suggested that porcine uterine development occurs in an ovary-independent manner before, and in an ovary-dependent manner after, 60 d of age [4, 5]. Before 60 d of age, Christenson et al. found that Meishan gilts achieved a superior endometrial gland development than white crossbred gilts [9]. Here, we further compared the uterine development between Meishan gilts and L×Y gilts during ovary-dependent development period (after 60 d of age), and found that Meishan gilts have increased endometrial gland area and uterine horn length than L×Y gilts. Thus, the superior uterine development of Meishan gilts than white crossbred L×Y gilts lasted from neonate to mature. In ovary-dependent development period, ovary-derived hormones estradiol and progesterone play crucial roles in endometrial glands development [5]. Recent evidences suggest that gut microbiota plays a role in the regulation of ovarian hormones estradiol and progesterone [12, 33, 34]. We found that Meishan gilts had a distinctive structure of gut microbiota and superior alpha diversity including observed species, Chao1, ACE, Shannon and Simpson compared with L×Y gilts under the same housing and feeding conditions. These conservative differences of gut microbiota between Meishan gilts and L×Y gilts indicated that host genetics can shape gut microbiota, which had been also reported in human [35]. Compared well with our results, Yuan et al. reported patients with endometriosis, a chronic and estrogen-dependent disease characterized by endometrial glands and stroma outside the uterus, had a disordered gut microbiota of reduced alpha diversity [36]. These increased alpha diversity indices had been also determined in Meishan sows whose reproductive performance were promoted by dietary crude fiber supplementation [10]. Further taxon analysis showed that Meishan gilts’ gut harbored more bacteria of phyla Actinobacteria Firmicutes, Fibrobacteres, Lentisphaerae, and Synergistetes but fewer bacteria of phyla Bacteroidetes and WPS-2 compared with L×Y gilts. Yang et al. also reported a similar difference of gut microbiota between Meishan sows and Duroc sows except Bacteroidetes [37]. These inconsistent results may because of the detect method (real-time PCR analysis vs. 16S rDNA high-throughput sequencing) or animal conditions (age etc.). Consistent with our results, Jiang et al. also determined an increased Bacteroidetes in fertility-improved Meishan sows by dietary crude fiber supplementation [10]. Among the changed bacteria at genus levels, the increased *Fibrobacter*, but decreased *Streptococcus* in Meishan gilts were comparable to the earlier reports [10, 38]. Taken together, our data firstly suggested that sexually mature Meishan gilts achieved superior endometrial gland area and uterine horn length than L×Y gilts, which may be regulated at least partly by their distinctive gut microbiota.

We then explored the association between gut microbiota and uterine development by transplanting the fecal microbiota of Meishan gilts to L×Y gilts. As reported in other FMT experiment [39, 40], FMT had a lasted shift-effect on the gut microbiota of recipient L×Y gilts and made them similar to that of the donor Meishan gilts. Interestingly, FMT from Meishan gilts increased endometrial gland area in recipient L×Y gilts. To further understand the association between the shifted gut microbiota and increased endometrial gland area, we profiled the metabolites of liver and plasma and found that FMT markedly shifted the metabolite profiles of both liver and plasma, and these FMT-induced differential metabolites from both liver and plasma enriched in steroid hormone biosynthesis pathway. Compared well with the results of metabolomics, we found that FMT increased estradiol concentrations, but decreased progesterone concentration in plasma during the trial dynamically. Ovary-derived steroid hormones of estradiol and progesterone play crucial roles in endometrial glands development [7]. Estradiol is a primary mitogen for uterine epithelium and can induce proliferation of both uterine luminal epithelium and glandular epithelium [7]. Progesterone reversely inhibit estradiol actions in stimulation of uterine epithelial proliferation through epithelial progesterone receptors [7, 13]. Therefore, FMT induced an increase of plasma estradiol concentrations, but a decrease of plasma progesterone concentration during our trial dynamically may account for its effect on promoting endometrial gland area. The elevated estradiol concentration but decreased progestogen in ovary tissue showed that FMT could regulate steroid hormones synthesis of ovary. Besides, estradiol and progestogen affect endometrial IGF-I expression and secretion [32]. In our study, FMT elevated IGF-1 concentration, and increased expression of *ESR1* gene in uterine tissue, suggesting that gut microbiota affect endometrial IGF-I expression and secretion by regulating steroid hormones estradiol and progestogen. CDH1 and FOXA2 are essential for endometrial gland differentiation and development [41, 42]. The elevated expression of both *CDH1* and *FOXA2* genes further confirmed that FMT promoted endometrial gland differentiation and development in recipient L×Y gilts. Steroid hormones regulate gene expression of *CDH1* during the peri-implantation period of pregnancy in pigs [43]. In ovariectomized mice, estradiol suppressed *Foxa2* mRNA expression at 48 h but not 4 h and 24 h after treatment [44]. This suppression effect of estradiol was sped up by co-treatment with progestogen [44]. However, the role of steroid hormones on the expression of *CDH1* and *FOXA2* genes during pre-puberty gilts remain unknown. Overall, FMT shifted the steroid hormones profiles, which may contribute to its effect on promoting endometrial gland development.

Given that gut microbiota gets involved in regulation of ovarian hormones estradiol and progesterone [12, 33, 34], we then identified the FMT-shifted bacteria. FMT increased Firmicutes, Fibrobacteres, *Bifidobacterium*, and *Fibrobacter*, but deceased Bacteroidetes. Nuriel-Ohayon et al. found that *Bifidobacterium* abundance increased in the gut during pregnancy in women and mice, and progesterone supplementation increases *Bifidobacterium* abundance in mice and *in vitro* [12]. Consistently, these FMT-shifted bacteria along with increased estradiol and progesterone had been also determined in Meishan sows whose reproductive performance had been promoted by dietary crude fiber supplementation [10], which suggested the potential interaction between gut microbiota and progesterone. More directly, gut microbiota gets involved in enterohepatic circulation of estradiol by encoding β-glucuronidase, which plays an important role in cleaving conjugated estradiol [38, 45]. The gut microbiome includes an ‘estrobolome’ that the aggregate of enteric bacterial genes whose products can cleave conjugated estradiol [45]. Notably, these FMT-shifted bacteria belong to the estrobolome. Taken together, FMT-shifted gut microbiota may promoted endometrial gland area by regulating steroid hormones. Furthermore, We found that FMT enriched KEGG functions of carbohydrate metabolism and digestive system. These function shifts of gut microbiota compared well with the increased SCFAs-producing bacteria, including Fibrobacter. In addition, these enrichment effects of FMT was confirmed by the increased concentrations of fecal SCFAs, including propionate and butyrate but not acetate. Although there is no direct evidence of SCFAs regulating endometrial gland development, the elevated fecal SCFAs get well along with the hyper-prolificacy of sows had been reported in Meishan sows and crossed white sows [10, 38, 46]. Recently studies indicated that these elevated SCFAs may promote sows’ prolificacy indirectly. Administration of SCFAs to mice alleviate stress-induced brain–gut axis alterations [47]. Kimura-Todani et al. also reported that acetate exerted an anxiolytic effect on the host [48]. However, we haven’t observed a significant increase of acetate concentration in distal colon, which may because of the acetate could be absorbed in colon [49]. In modern pig production system, sows suffer from postpartum stress even anxiety, which results in decreased maternal performance of lactating sows and increased pre-weaning piglet mortality [50, 51]. Given that the number of pigs weaned per sow per year is used to determine sows’ prolificacy [52], gut microbial metabolites SCFAs may contribute to the hyper-prolificacy of sows by ameliorating sows’ postpartum stress. Besides, the significant correlation between FMT-shifted gut microbes and uterine development-related indices further supports our hypotheses that gut microbiota contribute to endometrial gland development during the ovary-dependent period.

## Conclusion

In conclusion, this study presented that sexually mature Meishan gilts achieved a superior uterine development than L×Y gilts under the same housing and feeding conditions. Meanwhile, Meishan gilts harbored a distinctive gut microbiota that superior in alpha diversity when compared with L×Y gilts. Transplanting fecal microbiota from Meishan gilts made the gut microbiota of recipient L×Y gilts similar to that of the donors lastingly and promote endometrial gland development, simultaneously. The FMT-induced profiler shifts of steroid hormones which potentially driven by gut microbiota contribute to the promotion of endometrial gland development. Therefore, our data suggests that gut microbiota contribute to the endometrial gland development during the ovary-dependent period.

## Supplementary Information

**Additional file 1: Supplemental Table S1.** Ingredient composition and chemical analysis of the basal diet for tilapia.

**Additional file 2: Supplemental Figure S1.** Relative abundance of gut microbes in Meishan and LxY gilts.

**Additional file 3: Supplemental Figure S2.** FMT shifted the composition of gut microbiota in recipient LxY gilts.

## Data Availability

All data generated or analyzed during this study can be made available by the corresponding author upon reasonable request.

## Abbreviations

SCFAs: Short chain fatty acids
ELISA: Enzyme-linked immunosorbent assay
IGF-1: Insulin-like growth factor 1
EDTA: Ethylenediaminetetraacetic acid
ESR1: Estrogen receptor 1 gene
CDH1: Cadherin-1
FOXA2: Forkhead box protein A2
H&E: Hematoxylin and eosin
LC-MS: Liquid chromatography mass spectrometry
PICRUSt2: Phylogenetic Investigation of Communities by Reconstruction of Unobserved States 2
OTU: Operational taxonomic units
