# Supplementary material for: Gut microbiota contributes to the development of endometrial glands in gilts during the ovary-dependent period
Source: J Anim Sci Biotechnol. 2021 May 5;12:57. doi: 10.1186/s40104-021-00578-y (PMC8097987; doi:10.1186/s40104-021-00578-y)
Supplement: Supplementary file 1 — Additional file 1: Supplemental Table S1. Ingredient composition and chemical analysis of the basal diet for tilapia. [file 40104_2021_578_MOESM1_ESM.docx]

Supplemental Table S1 Ingredients and nutrients composition of the diet used in this trial^1^

| Ingredients | | Percent (%) | |  |
| --- | --- | --- | --- | --- |
| Corn | | 65.15 | |  |
| Wheat bran | 9.34 | |  |  |
| Soybean meal | 17.72 | |  |  |
| Rice bran meal | 3.99 | |  |  |
| CaCO_3_ | 0.8 | |  |  |
| CaHPO_4_ | 1.5 | |  |  |
| Salt | 0.34 | |  |  |
| DL-Methionine | 0.06 | |  |  |
| L-Lysine | 0.10 | |  |  |
| Vitamin premix^1^ | 0.5 | |  |  |
| Mineral premix^2^ | 0.5 | |  |  |
| Total | 100 | |  |  |
| Calculated nutrient composition |  | |  |  |
| Net energy (MJ/kg) | | 9.43 | | |
| Total Lys (%) | 1.08 | |  |  |
| Total Met (%) | 0.32 | |  |  |
| Total Met + Cys (%) | 0.60 | |  |  |
| Available phosphorus (%) | 0.68 | |  |  |
| Measured nutrient composition |  | |  |  |
| Crude protein | 15.69 | |  |  |
| Calcium (%) | 0.90 | |  |  |

^1^Vitamin premix provided per kg of diet: retinyl acetate, 10000 IU; cholecalciferol 2500 IU; dl-α-tocopheryl acetate, 50 IU; menadione, 5.0 mg; thiamin, 2.0 mg; riboflavin, 5.0 mg; pantothenic acid, 12.0 mg; pyridoxine, 10.0 mg; niacin, 30.0 mg; *d*-biotin, 0.2 mg; folic acid, 1.5 mg; cyanocobalamin, 0.05 mg; choline chloride 1500 mg.

^2^Mineral premix provided per kg of diet: FeSO_4_•7H_2_O, 498 mg; CuSO_4_•5H_2_O, 78.7 mg; MnSO_4_•5H_2_O, 110 mg; ZnSO_4_•7H_2_O, 440 mg; Na_2_SeO_3_, 0.66mg; KI, 0.4 mg.
