# Supplementary figures and images for: Gut microbiota contributes to the development of endometrial glands in gilts during the ovary-dependent period
Source: J Anim Sci Biotechnol. 2021 May 5;12:57. doi: 10.1186/s40104-021-00578-y (PMC8097987; doi:10.1186/s40104-021-00578-y)

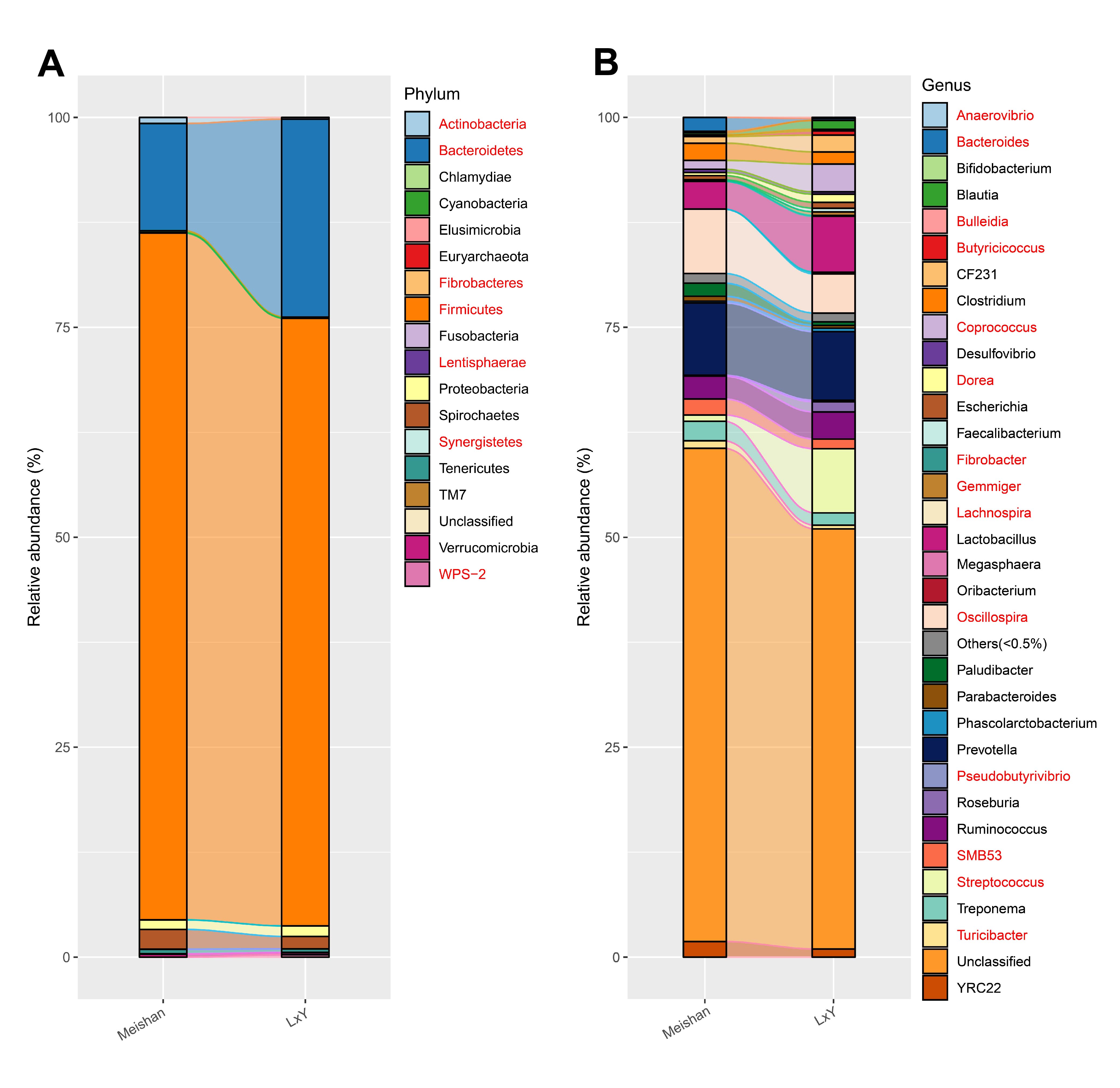

Supplement: Supplementary file 2 — Additional file 2: Supplemental Figure S1. Relative abundance of gut microbes in Meishan and LxY gilts. [file 40104_2021_578_MOESM2_ESM.jpg]

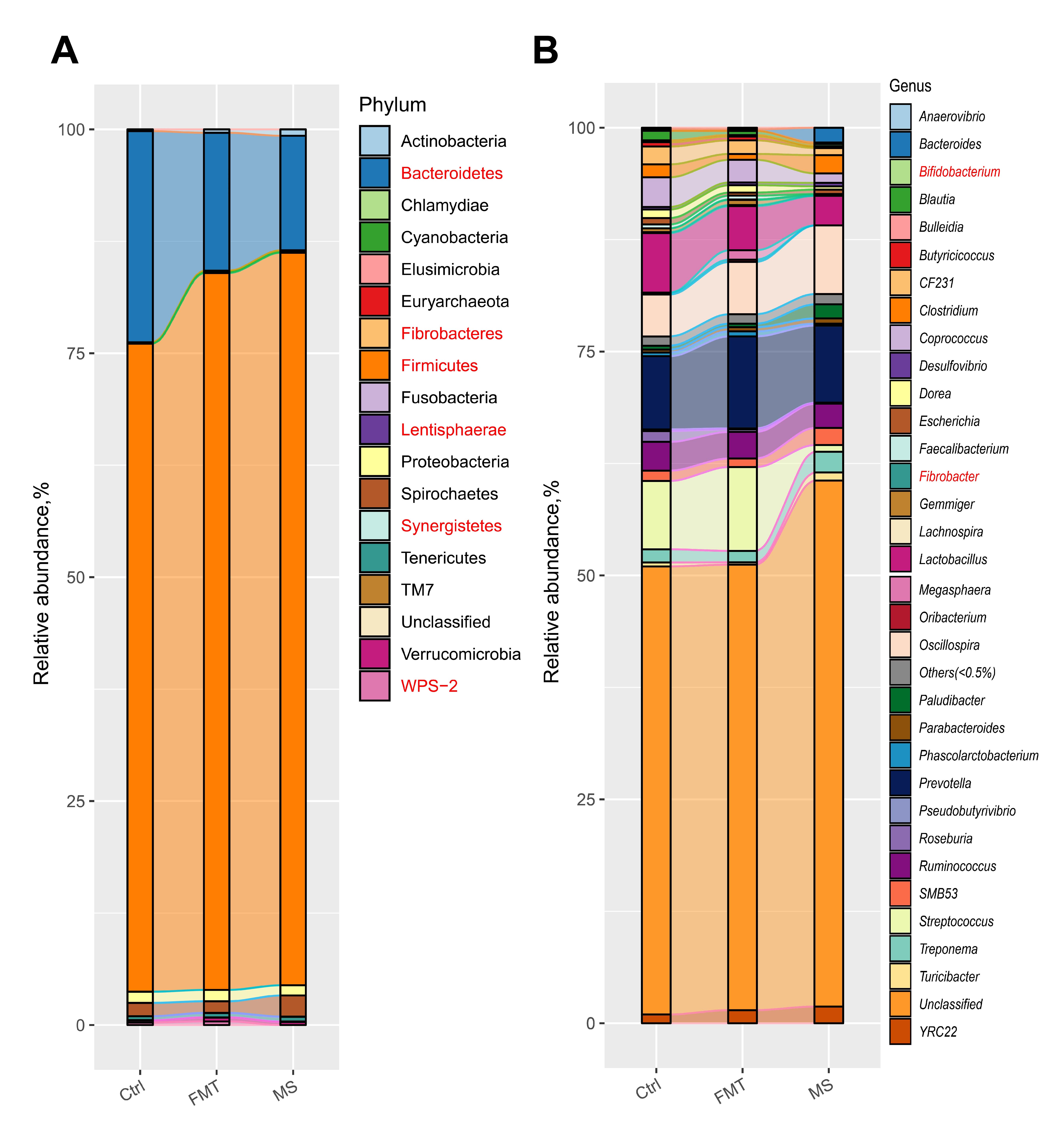

Supplement: Supplementary file 3 — Additional file 3: Supplemental Figure S2. FMT shifted the composition of gut microbiota in recipient LxY gilts. [file 40104_2021_578_MOESM3_ESM.jpg]
